# Evaluating few-shot prompting for spectrogram-based lung sound classification using a multimodal language model

**DOI:** 10.1371/journal.pdig.0001179

**Published:** 2026-01-07

**Authors:** Nicholas Dietrich, David McShannon, Mark F. Rzepka

**Affiliations:** 1 Temerty Faculty of Medicine, University of Toronto, 1 King’s College Cir, Toronto, Ontario, Canada; 2 Faculty of Engineering, McMaster University, Hamilton, Ontario, Canada; 3 Translational Medicine, Hospital for Sick Children Research Institute, Toronto, Ontario, Canada; Liverpool John Moores University - City Campus: Liverpool John Moores University, UNITED KINGDOM OF GREAT BRITAIN AND NORTHERN IRELAND

## Abstract

Traditional deep learning models for lung sound analysis require large, labeled datasets, whereas multimodal large language models (LLMs) may offer a flexible, prompt-based alternative. This study aimed to evaluate the utility of a general-purpose multimodal LLM, GPT-4o, for lung sound classification from mel-spectrograms and assess whether a few-shot prompt approach improves performance over zero-shot prompting. Using the ICBHI 2017 Respiratory Sound Database, 6898 annotated respiratory cycles were converted into mel-spectrograms. GPT-4o was prompted to classify each spectrogram using both zero-shot and few-shot strategies. Model outputs were evaluated against ground truth labels using performance metrics including accuracy, precision, recall, and F1-score. Few-shot prompting improved overall accuracy (0.363 vs. 0.320) and yielded modest gains in precision (0.316 vs. 0.283), recall (0.300 vs. 0.287), and F1-score (0.308 vs. 0.285) across labels. McNemar’s test indicated a statistically significant difference in performance between prompting strategies (p < 0.001). Model repeatability analysis demonstrated high agreement (κ = 0.76–0.88; agreement: 89–96%), indicating excellent consistency. GPT-4o demonstrated limited but statistically significant performance gains using few-shot prompting for lung sound classification. While current performance remains insufficient for clinical deployment, this prompt-based approach provides a baseline for spectrogram-based multimodal tasks and a foundation for future exploration of prompt-based multimodal inference.

## Author summary

Lung sounds, such as wheezes and crackles, can offer important clues about respiratory health. Traditionally, doctors use a stethoscope to listen for these sounds, but interpreting them accurately can be difficult and often requires experience. We wanted to explore whether a new type of artificial intelligence (AI), called a multimodal language model, could help identify different lung sounds by looking at visual representations of those sounds known as spectrograms. Specifically, we used a model called GPT-4o, which is designed to understand both images and text, and tested whether giving it a few examples of labeled lung sounds would help it perform better. We found that this few-example or “few-shot” prompting approach led to modest but meaningful improvements in how accurately the model could identify different types of lung sounds compared to giving it no examples at all. While the model’s current performance is insufficient for clinical deployment, our findings establish a foundational baseline, demonstrating that general-purpose AI tools can exhibit in-context learning to improve lung sound classification. This provides a direction for developing flexible and accessible AI support in resource-limited settings.

## Introduction

Lung sounds provide critical insights into pulmonary health and underlying disease. Traditionally, auscultation with a stethoscope has been the gold standard for bedside respiratory assessment [1–3]. However, advancements in technology have now enabled the use of electronic devices, such as digital stethoscopes and audio recorders, to capture and analyze respiratory sounds. These tools can be used to facilitate longitudinal monitoring, remote sharing, and integration of audio data into research pipelines for model development [4,5].

In addition, recent efforts have focused on developing machine learning (ML) models for automated lung sound classification, particularly in detecting adventitious sounds such as crackles and wheezes [6,7]. A common approach involves converting audio recordings into spectrograms and applying deep learning architectures, such as convolutional neural networks (CNNs), to build binary classifiers or detection models [8–10]. Despite promising performance, these models typically require substantial computational resources and extensive dataset-specific optimization, constraining their generalizability and limiting their use beyond specialized centres.

A potential alternative to CNNs for automated lung sound analysis is using general-purpose multimodal large language models (LLMs) to classify lung sounds [11]. Unlike traditional deep learning models, which rely on large, labeled datasets and require dedicated training for specific tasks, multimodal LLMs leverage pre-trained knowledge across diverse data formats, such as images, videos, and code. This flexibility may reduce the need for building task-specific architectures from scratch and instead shift the focus toward designing effective prompts. In this context, prompting strategies, which refer to structured instructions that shape how the model interprets and responds to an input, serve as a mechanism to optimize performance without explicit retraining. Such approaches may offer a scalable pathway for applying multimodal LLMs to defined tasks, like respiratory sound classification [12].

One such strategy is few-shot prompting, where a model is given a small set of labeled examples within the prompt to guide its output. Unlike zero-shot prompting, which provides no prior examples, few-shot prompting aims for models to infer patterns from limited contextual information. This strategy has been shown to improve LLM output accuracy for well-defined text-based tasks [12–14]. For example, prior research has demonstrated that few-shot prompting enhances performance in tasks, such as differential diagnosis generation and text classification [15]. However, whether this in-context learning extends to multimodal tasks, particularly in the classification of lung sound spectrograms, remains unexplored.

To our knowledge, no prior studies have evaluated the performance of a general-purpose multimodal LLM for lung sound analysis or investigated the impact of few-shot prompting in this context. Therefore, this study aims to address these gaps by assessing the ability of a multimodal LLM to classify lung sounds from mel-spectrograms and by comparing the baseline performance of few-shot versus zero-shot prompting.

## Materials and methods

### Population

This study utilized the previously validated International Conference on Biomedical and Health Informatics (ICBHI) 2017 Respiratory Sound Database [16], a publicly available dataset comprising lung sound recordings from 126 subjects across multiple clinical sites. The cohort included 46 females, 79 males, and 1 participant with missing sex data. Age distribution included 49 pediatric (<18 years), 22 adult (18–64 years), and 54 older adult (≥65 years) participants (1 participant with missing age data), with a median age of 60.0 years (interquartile range: 4.0–70.25). Audio recordings were collected using four types of electronic stethoscopes and microphones. The dataset contains 920 annotated recordings segmented into 6898 respiratory cycles (inspiration and expiration), labeled as “crackles” (n = 1864), “wheezes” (n = 886), “both” (presence of both crackles and wheezes; n = 506), or “normal” (absence of both crackles and wheezes; n = 3642) based on expert consensus.

### Spectrogram generation

Each audio file was segmented into individual respiratory cycles based on the start and end times provided in the ICBHI database [16]. Respiratory cycles were resampled to 44.1 kHz for uniformity, and a custom Python pipeline was developed to generate mel-spectrograms from these cycles, applying a Short-Time Fourier transform for frequency decomposition [17,18]. The mel-spectrograms were generated using 128 mel bands, a window size of 2048 samples, and a hop length of 512 samples, ensuring adequate time-frequency resolution. A viridis color filter was applied to enhance visual interpretability [19], and each spectrogram was structured with frequency (Hz) on the y-axis, time (s) on the x-axis, and amplitude in decibels (dB) to preserve critical acoustic features. Each spectrogram was saved in PNG format to maintain consistency in downstream analysis.

### Large language model selection

GPT-4o (model GPT-4o-2024-08-06) [20] was selected as the multimodal LLM for this study due to its native vision capabilities, and widespread use in prior multimodal research, including recent work demonstrating its ability to interpret non-medical spectrograms [21]. Developed by OpenAI, GPT-4o was trained on a diverse dataset that includes image, video, audio, and text modalities up to October 2023. The model was accessed via the OpenAI API, which allowed for precise control over temperature (set to 0 to minimize non-determinism), response length, and token constraints for deterministic outputs. Following common practice, the API was utilized in a stateless manner with data retention disabled, ensuring the model’s underlying weights did not change or “learn” during the study period [22]. The API’s batch processing capabilities enabled synchronous execution of prompts, maintaining uniformity across model queries while minimizing variability in responses.

### Prompt engineering and development

**Fig 1:**
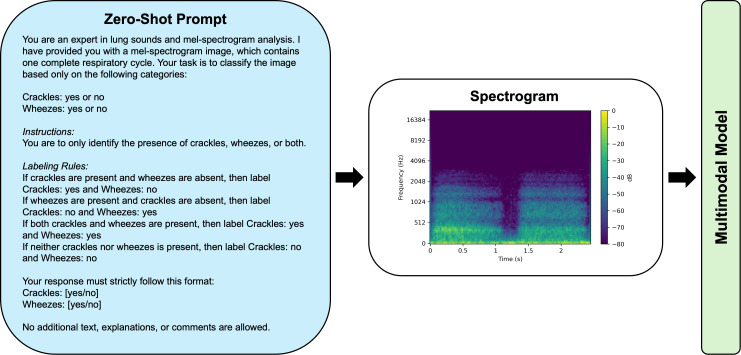
Zero-Shot Prompt Structure for Multimodal Model Lung Sound Classification.

**Fig 2:**
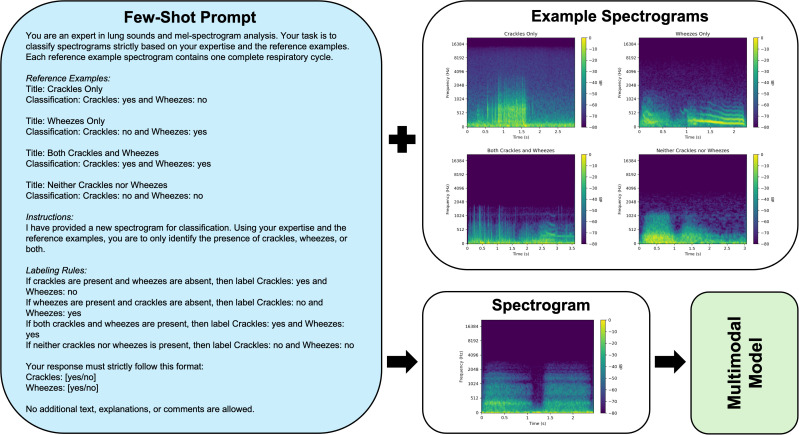
Few-Shot Prompt Structure with Labeled Reference Example Spectrograms for Multimodal Model Lung Sound Classification.

A standardized prompt structure was developed for both zero-shot and few-shot strategies. For the zero-shot strategy, GPT-4o was presented with the spectrogram and a predefined task question regarding lung sound classification, without any prior exposure to labeled examples (Fig 1). In contrast, the few-shot prompts incorporated examples of labeled reference spectrograms, providing the model with input-output pairs before classification of the spectrogram under evaluation (Fig 2). The reference spectrograms were derived from previously validated audio samples, representing crackles, wheezes, both, and normal sounds, obtained from an external dataset [23], and generated using the same preprocessing pipeline as the ICBHI database. This approach ensured a strict separation from the ICBHI testing set to prevent data leakage [24,25]. Few-shot exemplars were manually curated based on their spectral clarity and characteristic representation of the intended auscultatory category. The few-shot prompt design was informed by multimodal prompting strategies from prior studies, which have been shown to improve classification accuracy when contextual examples are provided [21,26].

### Data processing architecture

Each spectrogram was classified using the zero-shot and the few-shot prompts, with model settings and computational conditions identical across both prompting strategies. A synchronous processing pipeline was implemented to ensure that each spectrogram was sequentially processed, with each API request waiting for the previous request to complete before execution of the next. This controlled approach minimized variability and ensured that all classifications were performed under standardized conditions. The retrieved text outputs generated by GPT-4o were stored in .txt format, and a Python post-processing script was used to convert these outputs into a structure consistent with the ICBHI annotation format [16].

### Statistical analysis

The classification outputs from GPT-4o, using both zero-shot and few-shot strategies, were compared to the ground truth labels from the ICBHI dataset [16]. Performance metrics included accuracy, sensitivity (recall), specificity, precision (positive predictive value [PPV]), negative predictive value (NPV), and F1-score, computed as previously described [8,27]. These metrics were calculated both overall and on a per-label basis. Balanced accuracy (macro-averaged recall across the four classes) was also calculated to account for class imbalance. Raw classification counts for both prompting strategies were summarized using confusion matrices. Differences in overall classification performance between zero-shot and few-shot prompt strategies were assessed using McNemar’s test. Statistical significance was set at a two-sided p-value of 0.05.

For the per-label analysis, two analytical approaches were employed [16,27]. First, in the multiclass classification approach, the model was treated as a four-label classifier with mutually exclusive categories: only crackles, only wheezes, both, and normal. Second, in the binary classification approach, the model was treated as two independent binary classifiers, one for crackles (present/absent) and one for wheezes (present/absent), allowing for separate identification of each sound even when both were present in a single respiratory cycle. We also conducted a subgroup analysis for the “both” and “normal” ground truth labels to compare model performance on detecting crackles versus wheezes within these categories. This included calculating the frequency of each label prediction and evaluating differences between zero- and few-shot prompting using McNemar’s test for paired binary outcomes.

To assess model output repeatability, 10% of the dataset was randomly selected and re-evaluated using the same zero- and few-shot prompts under identical model conditions. This repeatability analysis was chosen over cross-validation because the study involves inference on a pre-trained LLM without model training or weight updates [28]. Classification labels for crackles and wheezes were compared between the initial and repeated outputs. For each condition, both Cohen’s kappa and percentage agreement were calculated to quantify the consistency between runs.

### Ethics statement

This study utilized the publicly available ICBHI 2017 Respiratory Sound Database [16], which comprises previously collected and anonymized recordings of human lung sounds. No new data were collected, and no direct interaction with human participants occurred. As the dataset is fully de-identified and publicly accessible, informed consent and ethical review were not required. Thus, in accordance with institutional research ethics guidelines, this study was determined to be exempt from ethics board review.

## Results

### Overall classification performance

Overall classification accuracy was 0.320 (95% confidence interval [CI]: 0.309–0.331) for zero-shot prompting and 0.363 (95% CI: 0.352–0.374) for few-shot prompting. For the zero-shot prompting, the model achieved a true positive (TP) count of 935, true negative (TN) of 1271, false positive (FP) of 2371, and false negative (FN) of 2321. These counts corresponded to a precision of 0.283 (95% CI: 0.267–0.298), recall of 0.287 (95% CI: 0.272–0.303), F1-score of 0.285, specificity of 0.349 (95% CI: 0.334–0.364), and NPV of 0.354 (95% CI: 0.338–0.369).

Using few-shot prompting, the model produced counts of 976 TP, 1529 TN, 2113 FP, and 2280 FN. Corresponding performance metrics included a precision of 0.316 (95% CI: 0.300–0.332), recall of 0.300 (95% CI: 0.284–0.315), F1-score of 0.308, specificity of 0.420 (95% CI: 0.404–0.436), and NPV of 0.401 (95% CI: 0.386–0.417). McNemar’s test revealed a statistically significant difference in performance (χ² = 86.47, p < 0.001), indicating that the few-shot strategy significantly outperformed the zero-shot strategy.

**Fig 3:**
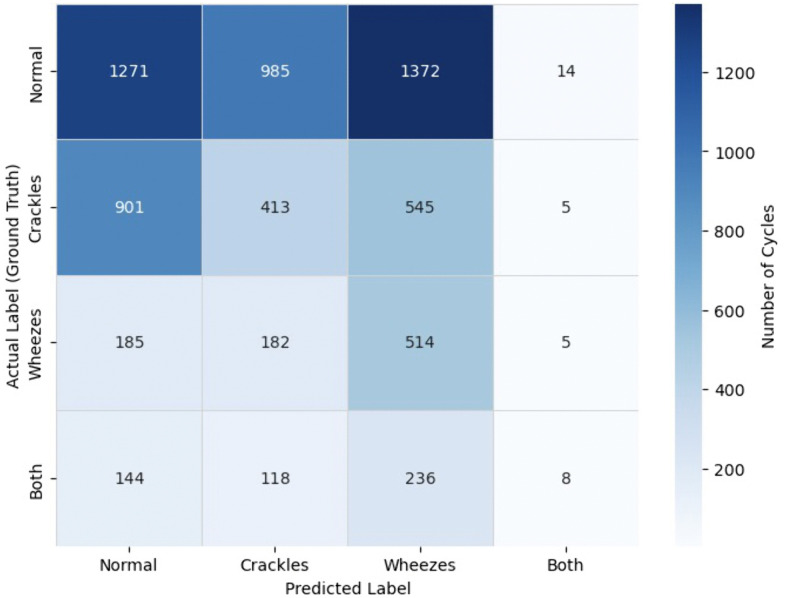
Confusion Matrix for Zero-Shot Prompting Strategy. The matrix displays the distribution of predicted versus ground truth labels across the four lung sound categories (normal, crackles, wheezes, both) for the zero-shot prompting strategy. Values represent the number of respiratory cycles classified into each category. Rows indicate ground truth labels, and columns indicate predicted labels.

**Fig 4:**
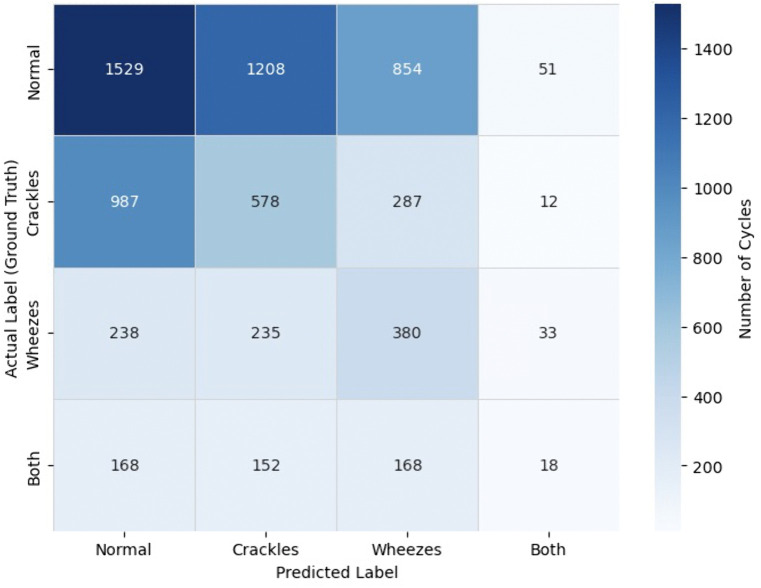
Confusion Matrix for Few-Shot Prompting Strategy. The matrix displays the distribution of predicted versus ground truth labels across the four lung sound categories (normal, crackles, wheezes, both) for the few-shot prompting strategy. Values represent the number of respiratory cycles classified into each category. Rows indicate ground truth labels, and columns indicate predicted labels.

For zero-shot prompting, balanced accuracy was 0.292, and few-shot prompting achieved a balanced accuracy of 0.299. Confusion matrices illustrating the distribution of correct and incorrect predictions across all four lung sound categories are provided for zero-shot (Fig 3) and few-shot (Fig 4) prompting strategies.

### Repeatability analysis

The repeatability analysis demonstrated Cohen’s kappa values of 0.88 and 0.78 for zero-shot crackles and wheezes, respectively, and 0.83 and 0.76 for few-shot crackles and wheezes, indicating substantial agreement across model runs. Agreement percentages were high for all comparisons, reported as 95.5% for zero-shot crackles, 89.3% for zero-shot wheezes, 92.6% for few-shot crackles, and 90.6% for few-shot wheezes. No statistically significant differences were observed in overall classification performance between the initial and repeated runs (p > 0.05), supporting the consistency and reliability of model predictions across repeated evaluations.

### Per-label classification performance

In the multiclass analysis, accuracy for the “only crackles” label was 0.603 (95% CI: 0.592–0.615) for zero-shot prompting and 0.582 (CI: 0.571–0.594) for few-shot prompting. For “only wheezes,” accuracy improved from 0.634 (95% CI: 0.623–0.645) in the zero-shot strategy to 0.737 (95% CI: 0.726–0.747) in the few-shot strategy. The “both” category showed high accuracy for both strategies, with 0.924 (95% CI: 0.918–0.931) for zero-shot and 0.915 (95% CI: 0.909–0.922) for few-shot prompting. Accuracy for the “normal” label was 0.478 (95% CI: 0.466–0.490) for zero-shot and 0.492 (95% CI: 0.480–0.504) for few-shot prompting.

Using the binary classification analysis, accuracy for detecting crackles was 0.563 (95% CI: 0.552–0.575) in the zero-shot strategy and 0.545 (95% CI: 0.533–0.557) in the few-shot strategy. For wheezes, accuracy improved from 0.628 (95% CI: 0.617–0.640) in the zero-shot strategy to 0.710 (95% CI: 0.700–0.721) with few-shot prompting. The remaining per-label performance metrics are reported in Table 1 for the four classes and Table 2 for the two binary classes.

**Table 1:** Multiclass performance metrics for each lung sound label (only crackles, only wheezes, both, normal) using zero-shot and few-shot prompting.

Label	Model Prompt	Accuracy (95% CI)	Precision (95% CI)	Recall (95% CI)	F1-Score	Specificity (95% CI)	NPV (95% CI)
Only Crackles	Zero	0.603 (0.592–0.615)	0.243 (0.223–0.264)	0.222 (0.203–0.240)	0.232	0.745 (0.733–0.757)	0.721 (0.709–0.733)
	Few	0.582 (0.571–0.594)	0.266 (0.247–0.285)	0.310 (0.289–0.331)	0.286	0.683 (0.670–0.696)	0.728 (0.715–0.741)
Only Wheezes	Zero	0.634 (0.623–0.645)	0.193 (0.178–0.208)	0.580 (0.548–0.613)	0.289	0.642 (0.630–0.654)	0.912 (0.904–0.921)
	Few	0.737 (0.726–0.747)	0.225 (0.205–0.245)	0.429 (0.396–0.461)	0.295	0.782 (0.772–0.793)	0.903 (0.895–0.911)
Both	Zero	0.924 (0.918–0.931)	0.250 (0.100–0.400)	0.016 (0.005–0.027)	0.030	0.996 (0.995–0.998)	0.927 (0.921–0.934)
	Few	0.915 (0.909–0.922)	0.158 (0.091–0.225)	0.036 (0.019–0.052)	0.058	0.985 (0.982–0.988)	0.928 (0.922–0.934)
Normal	Zero	0.478 (0.466–0.490)	0.508 (0.489–0.528)	0.349 (0.334–0.364)	0.414	0.622 (0.606–0.639)	0.461 (0.446–0.476)
	Few	0.492 (0.480–0.504)	0.523 (0.505–0.541)	0.420 (0.404–0.436)	0.466	0.572 (0.555–0.589)	0.469 (0.453–0.484)

Abbreviations: CI=confidence interval; NPV=negative predictive value.

**Table 2:** Binary classification performance metrics for crackles and wheezes using zero-shot and few-shot prompting.

Label	Model Prompt	Accuracy (95% CI)	Precision (95% CI)	Recall (95% CI)	F1-Score	Specificity (95% CI)	NPV (95% CI)
Crackles	Zero	0.563 (0.552–0.575)	0.314 (0.293–0.336)	0.230 (0.213–0.246)	0.265	0.738 (0.725–0.751)	0.647 (0.634–0.660)
	Few	0.545 (0.533–0.557)	0.332 (0.313–0.352)	0.321 (0.302–0.339)	0.326	0.663 (0.649–0.677)	0.651 (0.637–0.665)
Wheezes	Zero	0.628 (0.617–0.640)	0.283 (0.266–0.300)	0.548 (0.522–0.574)	0.373	0.648 (0.636–0.661)	0.850 (0.839–0.861)
	Few	0.710 (0.700–0.721)	0.332 (0.310–0.354)	0.430 (0.404–0.456)	0.375	0.781 (0.770–0.792)	0.844 (0.834–0.854)

Abbreviations: CI=confidence interval; NPV=negative predictive value.

On sub-analysis of the classification performance for the “both” label (where both crackles and wheezes were present in the ground truth), the model identified crackles in 126 zero-shot and 170 few-shot cases, and wheezes in 244 zero-shot and 186 few-shot cases. Only 8 instances had both crackles and wheezes correctly predicted in the zero-shot strategy, compared to 18 in the few-shot strategy. McNemar’s test revealed statistically significant differences in performance between zero- and few-shot strategies for both crackles (χ² = 27.191, p < 0.001) and wheezes (χ² = 38.679, p < 0.001).

For the “normal” label (where both crackles and wheezes were absent), the model correctly predicted absence of crackles in 2,643 zero-shot and 2,383 few-shot cases, and absence of wheezes in 2,256 and 2,737 cases, respectively. Both crackles and wheezes were jointly absent in 1,271 zero-shot and 1,529 few-shot cases. Again, McNemar’s test showed significant differences between zero- and few-shot prompting for both crackles (χ² = 140.337, p < 0.001) and wheezes (χ² = 344.395, p < 0.001).

## Discussion

To our knowledge, this study is the first to evaluate the classification of lung sound spectrograms using a general-purpose multimodal LLM and to compare few-shot and zero-shot prompting strategies in a respiratory sound context. Few-shot prompting produced marginal improvements in spectrogram-based classification performance, yielding statistically significant gains over zero-shot prompting across several metrics, including accuracy, specificity, and F1-score for the majority of lung sound labels. These findings align with recent work demonstrating that few-shot prompting can enhance performance on text-based medical tasks [25,26,29]. Despite these improvements, overall model performance remains insufficient for clinical deployment, positioning this work as an exploratory benchmark for visual in-context learning in this domain.

Although GPT-4o was evaluated on a large and clinically diverse dataset using a standardized preprocessing pipeline, the overall performance of both prompting strategies fell below clinically acceptable thresholds, with overall accuracy for zero-shot and few-shot strategies at 32.0% and 36.3%, respectively [8]. These values are lower than those reported for task-specific deep learning models trained on the ICBHI dataset [16], including CNNs, audio spectrogram transformer models, and other architectures, which typically achieve accuracy ranges of approximately 50 to 90% depending on preprocessing, model design, and class balancing techniques [8,27,30–32]. Human benchmark studies similarly report diagnostic accuracy between 55 and 90%, with crackles and wheezes consistently posing greater interpretive difficulty than normal breath sounds [31,33]. For comparison, in the multiclass setting, a random-chance classifier would achieve an accuracy of 25%; thus, the observed 32.0% (zero-shot) and 36.3% (few-shot) performance, while above random chance, underscores the limited discriminative ability of both strategies on this task. Consistent with these findings, multiclass balanced accuracy also remained low (0.292 for zero-shot vs. 0.299 for few-shot), indicating that explicitly adjusting for class imbalance does not materially change the overall performance profile. At the same time, the slight improvement with few-shot prompting suggests a small but measurable benefit in more evenly distributing predictions across classes, though this remains far from closing the gap with task-specific models.

Both prompting strategies showed notably poor performance in identifying lung cycles containing simultaneous crackles and wheezes, indicating that the multimodal LLM may struggle to integrate compound acoustic features. In the multiclass setting, recall for this category remained below 4%, and F1-scores were less than 10%. These findings suggest that when acoustic features overlap or co-occur with considerable variability, GPT-4o may be unable to separate individual sounds without task-specific training [34]. Furthermore, the inconsistent class-wise trends, such as the drop in crackles accuracy (from 60.3 to 58.2%) alongside a rise in wheezes accuracy (from 63.4 to 73.7%) with few-shot prompting, suggest that the effect of few-shot learning is not uniformly beneficial across all classes. This may be due to the more continuous and tonal nature of wheezes, which manifest in spectrograms as sustained horizontal patterns, features that may be more visually distinctive to a multimodal LLM. Crackles, on the other hand, are brief, discontinuous events whose sporadic nature may be less readily interpretable by a vision model without prior domain training [35]. In contrast, the “normal” class showed comparatively strong and consistent performance, likely due to the relative visual uniformity of spectrograms lacking adventitious sounds and the absence of unpredictable crackle or wheeze patterns within a respiratory cycle [35].

Taken together, our results suggest that GPT-4o shows limited but measurable responsiveness to few-shot prompting for spectrogram-based classification. Although its performance remained substantially lower than that of task-specific deep learning models–and well below the gains typically reported for text-based tasks [26]–the modest gains observed with contextual examples indicate a small degree of visual in-context learning without domain-specific training [26]. Considering these findings, exploration of other prompt-based approaches—for example, varying the number or visual characteristics of exemplars, or considering techniques such as chain-of-thought or retrieval-augmented prompting—could help clarify the extent to which prompting strategies influence model performance [36–39]. In parallel, broader evaluation across diverse patient populations, real-world or noisy recordings, and additional adventitious lung sounds such as rhonchi and stridor may help clarify how these models behave across a fuller spectrum of pulmonary acoustics [40–42].

Beyond technical performance, translation of multimodal LLMs into clinical respiratory assessment tools requires addressing significant practical, regulatory, and ethical hurdles. As no LLMs are currently cleared for standalone diagnostic use, robust “human-in-the-loop” oversight is critical for ensuring safety and accountability as these technologies advance toward clinical evaluation and potential deployment [25,43]. This approach aligns with existing regulatory precedents for AI-augmented digital auscultation tools, where authorization is limited to assistive interpretation requiring clinician oversight [44–46]. Additional concerns include liability arising from model errors or “hallucinations”, particularly regarding who bears responsibility if incorrect or misleading outputs contribute to diagnostic delay, inappropriate management, or missed pathology. These issues are further compounded by uncertainty about sustaining reliable performance across diverse patient populations and variable recording conditions, as well as the need for mechanisms to monitor and detect performance drift over time [12,47,48]. The susceptibility of LLMs to adversarial inputs, subtle prompt manipulations, and context-dependent errors adds another layer of complexity that must be carefully examined before clinical adoption [49,50]. Advancing toward regulated medical use will also require clear pathways for accountability and validation. While formal regulatory structures for generative AI and multimodal models are limited, emerging frameworks, such as Health Canada’s pre-market guidance for ML-enabled medical devices [51] and the FDA’s draft guidance for AI-enabled device software function [52], provide an early structure for safety documentation and risk mitigation. Ultimately, meaningful clinical integration requires large-scale real-world validation studies, standardized evaluation protocols, and a focus on equitable access to ensure that AI-augmented respiratory assessment tools are usable across both high- and low-resource environments [53–55].

This study was not without its limitations. First, although spectrograms were generated from the ICBHI 2017 dataset using a standardized preprocessing pipeline to ensure visual consistency, this transformation may not preserve all acoustic nuances present in the original recordings, particularly subtle transient features or sounds masked by background noise [16]. Nonetheless, such noise and variability reflect real-world conditions and support generalizability. Second, while the zero- and few-shot prompts followed previously validated approaches and were applied consistently across all 6,898 cases, model outputs remain inherently sensitive to prompt formulation, and alternative prompt structures could yield different results [37]. Lastly, this study evaluated the performance of a single multimodal LLM, which, although GPT-4o is a popular and well-studied foundation model, may not reflect the performance of smaller, domain-specific, or medically fine-tuned models [56]. Future work should benchmark performance across emerging multimodal architectures to contextualize these findings within the rapidly evolving LLM landscape.

## Conclusion

Our study demonstrates that few-shot prompting generally achieves marginal improvements over zero-shot prompting for mel-spectrogram-based lung sound classification using a general-purpose multimodal LLM. Although overall performance remains far below that of task-specific deep learning models and insufficient for clinical use, this approach provides a baseline for visual in-context learning and foundation for subsequent refinement. Further work should focus on optimizing prompting strategies and evaluating performance across more diverse, clinically representative datasets.
